# Case of Poisoning by the Extract of Belladonna

**Published:** 1848-07

**Authors:** 


*Case of Poisoning by the Extract of Belladonna.*—The following case of poisoning by Belladonna, occurred at Black Rock on the 15th instant:—Charles Stevens, boarding at Tilden's Tavern, procured from an apothecary of this city, what purported to be a quarter of a pound of Dandelion, on a prescription given by Dr. L. P. Dayton, of Black Rock. On his return the medicine was prepared by adding to it about a pint and a half of water, and some loaf sugar. Of this the patient took, as nearly as could be ascertained, between two and three ounces, making from 120 to 180 grains of the extract. He shortly complained of vertigo, impaired vision, etc., and Dr Dayton was called to visit him. When Dr. D. first saw him, he was able to give, what appeared at the time a coherent account of the circumstances connected with procuring the medicine, but the account was incorrect, according to his subsequent statement. Dr. D. finding the pupils dilated, in connection with the other symptoms, inferred that the medicine was Belladonna, and prescribed, at once, an emetic of Ipecucnanha, and the sulphate of zinc, which operated satisfactorily. The surface and extremities being cold, sinapisms were ordered. He shortly lapsed into a semi-lethargic state. Dr. White and Dr. Flint saw him, by request of Dr. Dayton, about five hours after the medicine had been taken. He then lay apparently in tranquil sleep, without stertor, and respiration quite natural. Pulse 86, and otherwise normal. Countenance presented a bloated appearance, and the expression was abolished, as in profound intoxication. On raising the eye lids the pupils were largely dilated, a ring of iris being alone visible. When shaken, and his name loudly pronounced, he opened his eyes with a wild, staring expression. He replied to questions incoherently, frequently in a ludicrous manner. Occasionally he extended his hand as if feeling for something. Vision was manifestly nearly, if not quite absent. On directing him to drink some water, great difficulty of deglutition was apparent, and the effort excited some retching. The act of swallowing was frequently repeated while lying in a somnolent state. The tongue was furred, and on examination, the throat presented a reddened aspect, the tonsils being somewhat swelled. We remained with the patient a couple of hours, at the end of which time he could be roused with greater facility, the other symptoms remaining the same. Dr. D. had administered castor oil after the emetic, and before our arrival. During our stay, carbonate of ammonia in solution was given frequently, and continued through the night. He progressively improved after our departure, and by morning he had recovered his intelligence, and the other symptoms had mostly disappeared. Mr. Tilden, the keeper of the tavern, while the medicine was preparing, tasted of it repeatedly, and remarked at the time, that the taste was unlike the extract of dandelion which had been before prescribed in his family. He also was affected with giddiness, and staggered in walking as if intoxicated. His pupils, at the time of our visit, were considerably dilated, and his vision imperfect.

On examination of the medicine, it was found to differ in its sensible properties from Taraxacum, and resembled Belladonna; and on applying it around one of the eyes of a young man who was present, the pupil soon became largely dilated.

The pot containing the extract, had upon it the following label:—

EXTRACT OF
DANDELION
*Leontodon Taraxacum.*
D. J. H.

*Prepared in the United Society, at Mt. Lebanon, N. Y. Put up in jars to suit purchasers, and sold, wholesale and retail, by BROTHERS HIBBARD, general agents for the Shakers.* 96 *John Street, New York.*

Another pot, with the same label, from the same invoice of 25 or 30 pots, was examined, and appeared to be also Belladonna. How much of it is abroad it is impossible to say, but the above suggests the importance of caution in purchasing or using extracts thus labelled, without careful examination.

This accident may serve to illustrate the necessity for subjecting domestic, as well as imported remedies, to official inspection, and compelling druggists and apothecaries to sell only those medicines which have undergone proper examination. The public, by and by, will perceive the importance of such a measure for their own safety.*

* Dr. Dayton has informed us, since this article was written, that the iris of the patient is still dilated, (18th) and that he appears listless, and absent-minded—but is daily improving.

